# Development and Evaluation of a 3D Motion Capture Model for Upper Extremity Kinematics During Wheelchair Maneuvering in Individuals with Spinal Cord Injuries: A Pilot Study

**DOI:** 10.3390/bioengineering12060648

**Published:** 2025-06-12

**Authors:** Lina Bunketorp Käll, Gudni Rafn Harðarson, Erik Tullin, Ann-Sofi Lamberg, Roy Tranberg, Johanna Wangdell

**Affiliations:** 1 Department of Occupational Therapy and Physiotherapy, Sahlgrenska University Hospital/Mölndal, 431 80 Mölndal, Sweden; erik.g.johansson@vgregion.se (E.T.); ann-sofi.lamberg@vgregion.se (A.-S.L.); johanna.wangdell@gu.se (J.W.); 2 Department of Health and Rehabilitation, Institute of Neuroscience and Physiology, Sahlgrenska Academy, University of Gothenburg, 405 30 Gothenburg, Sweden; 3 Department of Orthopaedics, Institute of Clinical Sciences, University of Gothenburg, 413 45 Gothenburg, Sweden; gudni.hardarson@gu.se (G.R.H.); roy.tranberg@gu.se (R.T.); 4 Department of Hand Surgery, Institute of Clinical Sciences, Sahlgrenska Academy, University of Gothenburg, 431 80 Mölndal, Sweden

**Keywords:** motion capture, spinal cord injury, wheelchair, performance, reliability testing

## Abstract

Spinal cord injury (SCI) often necessitates the use of a manual wheelchair, which can overload the shoulders and contribute to upper extremity (UE) pain. Currently, no standardized methods exist to assess UE kinematics during wheelchair propulsion. This study aimed to develop and evaluate a marker-based motion capture model for analyzing UE movement during wheelchair use, with a secondary goal of assessing test–retest reliability. The study was conducted in two phases: (1) development of the motion analysis model and (2) reliability testing. Eleven participants with SCI were included. Reliability was assessed using intraclass correlation coefficients (ICCs) across 15 movement parameters, including total range of motion and minimum and maximum movement values. The model demonstrated good test–retest reliability. For minimum movement, 12 of 15 parameters were significant (ICC = 0.681–0.965). For maximum movement, 13 of 15 were significant (ICC = 0.726–0.981). For total range of motion, 12 of 15 showed significant reliability (ICC = 0.596–0.952). In conclusion, the motion capture model showed promising reliability for assessing UE kinematics during wheelchair maneuvering in individuals with SCI. However, due to the small sample size, further research is needed to validate and refine the model.

## 1. Introduction

A spinal cord injury (SCI) has a profound impact on a person’s life, affecting motor, sensory, and autonomic nervous system functions [1]. Paralysis resulting from an SCI is classified as either paraplegia—affecting mobility in the lower half of the body while sparing the arms—or tetraplegia, which involves impairment of both the upper and lower extremities [2]. Many individuals with an SCI lose their ability to walk and become dependent on wheelchairs for mobility [3]. This reliance places significant strain on the upper extremities, which are not anatomically designed to sustain such daily mechanical loads. Consequently, individuals with an SCI who use manual wheelchairs experience higher rates of shoulder pain and dysfunction compared to able-bodied individuals [4,5,6]. The high incidence of shoulder pain in this population is multifactorial. One commonly suggested cause [7,8] is the overuse of the shoulder joint, which must compensate for the lost function of the hip joint during daily mobility. To effectively prevent and treat shoulder dysfunction in individuals with SCI, a thorough understanding of upper extremity (UE) biomechanics during wheelchair propulsion and daily tasks is essential [9]. Shoulder kinetics are complex, involving intricate movements that are difficult to observe and standardize through visual assessment alone. This presents a challenge in evaluating treatment outcomes. Therefore, objective measures of UE and shoulder kinetics are needed to develop effective therapeutic strategies that promote long-term independence and mobility.

Previous studies have examined UE motion during wheelchair maneuvering using 3D kinematics in individuals with SCIs [10,11,12,13]. These studies have aimed to analyze joint movements and biomechanical loads to better understand the mechanics of manual wheelchair propulsion. Methods often involve motion capture systems with reflective markers placed on key anatomical landmarks, allowing for the detailed tracking of limb movements. The results from these studies have contributed valuable insights into shoulder and arm kinematics, highlighting movement patterns and potential risk factors for overuse injuries. However, a significant limitation of these studies is the lack of standardized methodologies. Many do not provide sufficient details on marker placement, data processing, or analysis techniques, making it difficult to replicate findings or compare results across studies. This inconsistency hinders the ability to draw firm conclusions about UE kinematics during wheelchair propulsion.

This gap in knowledge underscores the need for more standardized and reproducible research methods to accurately assess UE motion in wheelchair users. Addressing this issue would provide more reliable insights into movement patterns and injury risk, ultimately supporting the development of improved rehabilitation strategies and assistive technologies. To the best of our knowledge, the kinematics of the shoulder during manual wheelchair propulsion has not been thoroughly described. There is a notable lack of studies examining UE kinematics in an open environment, free from treadmills or test benches. While some studies have analyzed shoulder and UE kinematics, the complex nature of forearm motion during wheelchair maneuvering has not, to our knowledge, been thoroughly examined.

To address this knowledge gap, we designed a methodological study to analyze UE kinematics during wheelchair propulsion in individuals with SCIs. A more comprehensive understanding of upper and lower extremity kinematics could provide a valuable framework for evaluating treatment effects, guiding rehabilitation strategies, and optimizing clinical decision-making. Additionally, such insights could play a crucial role in determining the most appropriate treatment options and informing surgical decisions for individuals experiencing shoulder-related complications. The primary aim of this study was to develop a marker-based motion capture model to analyze UE kinematics during manual wheelchair propulsion in individuals with SCIs. The secondary aim was to assess the model’s intra-rater reliability.

## 2. Materials and Methods

### 2.1. Study Design and Participants

This study was designed as a two-phase methodological investigation [14]: Phase 1: Development and adaptation of a marker-based motion capture model to assess UE kinematics during manual wheelchair propulsion. Phase 2: Evaluation of the test–retest reliability of kinematic measurements using the three-dimensional motion capture model. The inclusion criteria were (1) age ≥ 18 years; (2) history of SCI for ≥ 1 year; and (3) dependence on a manually propelled wheelchair for mobility. The exclusion criteria were (1) any condition other than SCI necessitating the use of a manual wheelchair and (2) the use of an electrically powered wheelchair or power-assisted wheels. Ethical approval was obtained from the regional ethics committee (approval number 239-18). All participants were fully informed about the study and provided written informed consent. For the test–retest trials, a snowball sampling method was employed. Initially, patient organizations supporting individuals with spinal cord injuries in the Gothenburg region were contacted [15]. A total of 20 individuals were invited to participate based on their availability and willingness to take part. The final study group consisted of 11 individuals with SCIs from the Gothenburg region, including 10 participants with paraplegia and 1 with tetraplegia. Demographic and clinical characteristics are presented in Table 1. All participants used their own fixed-frame lightweight wheelchairs (Panthera^®^, Stockholm, Sweden). None of the participants used removable footrests, and all had properly inflated tires and maintained a normal seated position.

### 2.2. Data Collection

**Figure 1:**
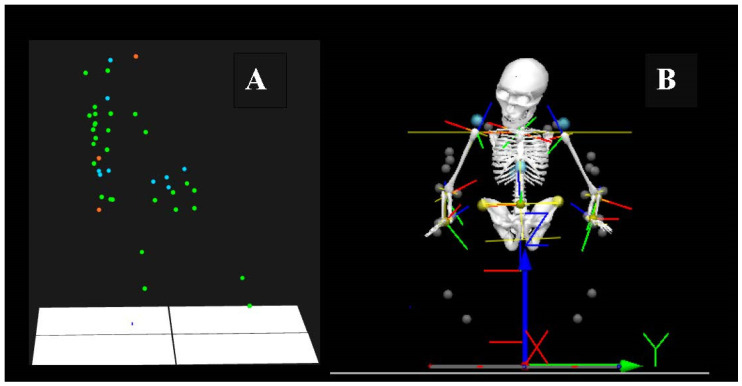
(**A**) Markers in QTM Track Manager (Qualisys, Gothenburg, Sweden); (**B**) 3D marker set in Visual3D (C-Motion, Germantown, MD, USA).

A three-degrees-of-freedom (3-DOF) model was developed for this study. The model utilized 36 markers positioned on the subject’s trunk, head, arms, and wheelchair (Table 2, Figure 1A,B). Marker placement was based on international recommendations from the Collaborative Upper Limb Pain Studies (CULP-SCI) and the International Society of Biomechanics, ensuring standardized and reliable kinematic measurements [16,17]. Calibration was performed at the start of each day using an L-frame for reference and a dual-marker wand for camera localization. Each calibration session captured 10,000 frames over a two-minute period. Calibration was deemed acceptable if the average residual error for any camera did not exceed 1 mm.

### 2.3. Experimental Setup and Test Protocol

**Figure 2:**
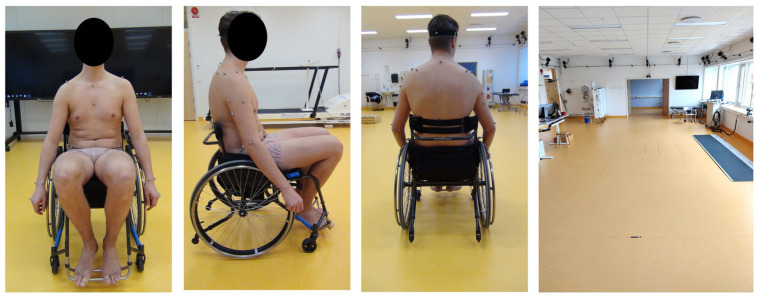
Marker placement and laboratory setup used during the experimental procedures.

The gait laboratory was equipped with 16 infrared (IR) cameras from the Qualisys Oqus series (Qualisys^®^, Gothenburg, Sweden) and two high-speed video cameras (Qualisys^®^, Gothenburg, Sweden). The infrared cameras recorded at 240 frames per second for both trials. The test–retest assessment was conducted on the same day, with a mandatory one-hour break between sessions. Participants were instructed to have a bare trunk and wear loose-fitting pants. If preferred, they could wear a tank top with narrow shoulder straps. A total of 36 ball-shaped, 12.5 mm (mm) reflective markers were used to track the motion of the participant and wheelchair. Thirty-two markers were attached to predefined anatomical locations on the upper body according to international recommendations from Collaborative Upper Limb Pain studies (CULP-SCI) and the International Society of Biomechanics [16,17]. Four additional markers were placed on the wheelchair to capture its motion in the laboratory. Figure 2 illustrates the marker placement and the laboratory setup used for the experimental procedures.

The markers reflected infrared light from the cameras, and the 16-camera setup ensured the optimal visibility of the markers throughout the wheelchair maneuvering tasks. Marker placement was performed by the same physiotherapist, who was experienced in SCI rehabilitation but new to motion capture analysis, ensuring consistency in application.

A laboratory technician was present during all measurements. The test was conducted on a 15 m long track, which previous studies have indicated is sufficient for assessing shoulder kinematics over time [18]. Each task was performed three times. Before the measurement, a static trial was conducted, in which participants assumed a standardized position: seated with locked wheels, elbows flexed to 90 degrees, arms close to the body, and hands pointing forward with thumbs up. After the static trial, marker visibility was verified before proceeding to dynamic trials. For the test–retest assessment, a mandatory one-hour break was implemented between trials to allow for the resolution of any skin irritation caused by marker application.

### 2.4. Kinematic Data Handling

Motion data was recorded using the Qualisys Track Manager software (https://www.qualisys.com/software/qualisys-track-manager/, Qualisys AB, Gothenburg, Sweden). Following data collection, raw motion data was manually processed by tracking markers placed on anatomical landmarks. A prediction error threshold of 25 mm was applied for marker tracking. The tracked motion data was then converted into .3cd files and transferred to Visual 3D (https://www.has-motion.ca/, HAS-Motion^®^, Kingston, ON, Canada) for segment building and further analysis. To minimize motion artifacts while preserving movement accuracy, a low-pass filter of 10 Hz was applied to the marker data. Joint angle calculations were performed between segments consequently using the proximal segment as the reference segment, and the processed motion data was exported as an American Standard Code of Information Interchange (ASCII) file for statistical analysis.

### 2.5. Data Analysis

The three-degrees-of-freedom (3-DOF) model was used to calculate data for shoulder abduction and shoulder flexion. However, due to its limitations in calculating internal rotation, the 3-DOF model was not used for assessing forearm pro-supination. Instead, the planar angle was calculated between a vector passing through the medial and lateral epicondyle markers and a vector passing through the medial and lateral wrist markers. This approach provided a more accurate representation of forearm rotation.

### 2.6. Data Output

To normalize the motion data of wheelchair propulsion, each trial was divided into propulsion cycles. Each cycle consists of two distinct phases [19]: 1. Push phase: This phase begins when the hand makes contact with the push rim, with the forearm positioned at its furthest point from the front of the wheelchair. It continues until the hand disengages from the push rim, at which point, the forearm reaches its maximum distance from the C7 vertebra. 2. Recovery phase: This phase starts when the hands disengage from the push rim and continues as the upper extremities swing forward until they make contact with the push rim again, marking the start of the next push phase. This segmentation allows for a standardized analysis of UE kinematics during manual wheelchair propulsion.

### 2.7. Statistical Analyses

Statistical analysis was conducted using R (R Project, version 4.4.0). Intraclass correlation coefficients (ICCs) were calculated for the minimum, maximum, and total range of motion using a two-way mixed-effects model, assessing absolute agreement with 95% confidence intervals. For total range of motion, the range for each strike across all trials was determined by subtracting the minimum value from the maximum value. A mean range of motion was then calculated, and statistical analysis was performed as described above. The same procedure was applied to minimal and maximal motion, where the mean maximal and mean minimal values were calculated across all strikes at the first and second assessments.

## 3. Results

**Figure 3:**
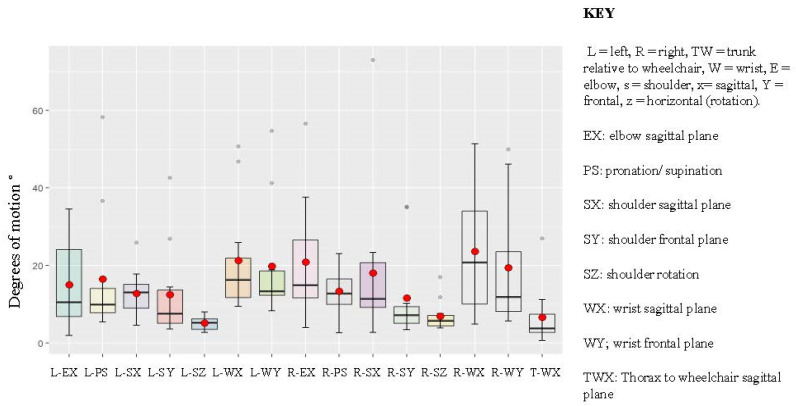
Boxplots depicting the maximum deviation from the average across three test–retest trials. The black line represents the median, gray dots indicate extreme values, and red dots denote the mean.

Figure 2 illustrates the maximum difference in motion between the test and retest assessments, averaged across three trials. Movements in the frontal plane demonstrated greater stability, as reflected in the distribution and shape of the box plots in Figure 3, compared to movements in the sagittal and horizontal (rotational) planes.

The results of the test–retest reliability assessment are presented in Table 3, Table 4 and Table 5. The ICC values represent the mean intraclass correlation coefficients calculated across all repeated trials for each motion parameter. For each participant, the maximum, minimum, and total range of motion values were averaged across three trials per test session before calculating the ICC for test–retest reliability. These mean ICC values, along with their 95% confidence intervals, reflect the overall consistency of measurements between the first and second sessions. When calculating ICCs for the total range of motion, 12 out of 15 motion parameters demonstrated significant reliability, with ICC values ranging from 0.596 to 0.952 (Table 3).

For the minimum range of motion, the ICC was significant for 12 motion parameters, with values ranging from 0.681 to 0.965 (Table 4). Similarly, for the maximum range of motion, 13 significantly correlated parameters exhibited ICC values between 0.726 and 0.968 (Table 5). The test–retest analysis indicates that motions in the horizontal plane (rotation and supination/pronation) show lower reliability compared to those in the frontal and sagittal planes. This trend is particularly evident in the analysis of minimum (Table 4) and maximum motions (Table 5). Overall, most of the analyzed motions demonstrate good to excellent reliability, according to established standards for reporting ICC values [20].

## 4. Discussion

A standardized motion capture model for assessing UE kinematics during wheelchair propulsion was developed and demonstrated to be an effective tool for quantifying UE movements in individuals with SCI. The 3-DOF model proved to be reproducible, and when combined with planar angle calculations, it allowed for a detailed visualization of the complex kinematics of the UE. Test–retest reliability analysis indicated that motions in the horizontal plane exhibited lower correlation compared to those in the frontal and sagittal planes. This outcome was expected, as even slight adjustments in shoulder positioning trigger a chain of compensatory movements throughout the arm during wheelchair maneuvering. For instance, an increase in shoulder abduction leads to greater internal shoulder rotation, followed by increased dorsiflexion of the hand. Consequently, minor variations in shoulder movement can result in amplified changes in the horizontal plane, contributing to lower ICC values.

### 4.1. Methodological Considerations

To extract data on pronation and supination movements—representing the internal rotation of the forearm—we employed a novel approach by calculating planar angles rather than relying on degrees-of-freedom models. Through test–retest trials and the manual verification of motion curves, we confirmed that the subjects exhibited similar movement patterns. The ±1 standard deviation for pronation–supination testing remained within a reasonable range considering that the normal range of motion for this movement is approximately 150–160°. This allows for greater flexibility in adjustment during maneuvering [21].

Previous research has investigated upper extremity kinematics during manual wheelchair propulsion, often focusing on shoulder, elbow, and wrist joint angles using standard motion capture techniques. For instance, studies by Boninger et al. [17] and Mulroy et al. [22] characterized propulsion biomechanics in individuals with spinal cord injuries (SCIs), highlighting the high mechanical demands placed on the glenohumeral joint. Compared to these studies, the current model offers a robust and standardized approach to quantifying upper limb kinematics, with a particular emphasis on reproducibility and practical implementation in clinical research settings. It aligns with the existing literature in reporting shoulder and elbow angle trajectories and complements previous findings by offering a tool that can be consistently applied across different labs and populations. In terms of joint angle assessment, the model is best suited for quantifying gross joint motions of the upper limb in the sagittal and frontal planes, including shoulder flexion/extension and abduction/adduction, elbow flexion/extension, and wrist flexion/extension.

The motion capture model developed in this study has several potential real-world applications. In rehabilitation settings, it can be used to monitor patient-specific propulsion patterns and detect maladaptive strategies that may predispose individuals to overuse injuries. By enabling detailed kinematic profiling, the model may support the development of individualized rehabilitation strategies that aim to optimize propulsion efficiency while minimizing joint strain. Additionally, the model could serve as a baseline for evaluating outcomes following surgical interventions, such as tendon transfers, by quantitatively assessing pre- and post-operative changes in upper limb function. Its reproducibility also makes it a strong candidate for use in longitudinal studies tracking functional recovery or deterioration in SCI populations. With further integration into clinical workflows, this model may support objective decision-making and facilitate personalized treatment planning.

An interesting observation from our results was the presence of asymmetries between left and right upper extremity joint angles, which was not explicitly discussed in the original manuscript. These asymmetries may stem from a combination of participant-specific factors and methodological considerations. Functionally, most manual wheelchair users demonstrate hand dominance and often favor one arm for propulsion and maneuvering, which can result in long-term asymmetrical movement patterns and muscle development. Such asymmetries have been previously reported in wheelchair users [17] and may be further exacerbated by postural adaptations or compensatory strategies unique to each individual’s injury level, strength, or range of motion.

However, it is also possible that part of the observed asymmetry is attributable to limitations in marker placement or the inherent challenges of measuring joint kinematics, particularly in individuals with altered muscle tone or body morphology following SCI. While every effort was made to ensure consistent and symmetrical marker placement, small deviations can affect the calculated joint angles. Future studies should explore the clinical significance of these asymmetries in greater depth, potentially using electromyography or imaging-based validation methods to distinguish between true biomechanical asymmetry and methodological artifacts.

### 4.2. Limitations and Future Work

This study has some limitations, including a small sample size, which should be considered when interpreting the results. However, a key strength is that the developed model is based on well-established technology and standardized marker placement, ensuring reliability and consistency. This study proposes a model for assessing UE kinematics during wheelchair maneuvering. Utilizing a stable and validated model facilitates its future application in evaluation and intervention studies. Clinically, this model could be used to assess prescribed treatments or post-surgical outcomes by objectively measuring movement patterns and kinematics. Additionally, it may aid in decision-making prior to treatment interventions. Future research should include larger, more diverse participant groups to further evaluate the model’s stability.

While the current model provides a reliable and reproducible method for assessing upper extremity kinematics during wheelchair propulsion, there are opportunities for further technological advancement. One promising direction is the integration of machine learning algorithms to automate data processing and improve the detection of joint centers and motion patterns, which could reduce operator dependency and improve efficiency. Additionally, markerless motion capture systems—though currently less precise for fine upper limb movements—are rapidly evolving and may, in the future, offer a more user-friendly and accessible alternative, especially in clinical or community settings where traditional systems are impractical. Incorporating such technologies could expand the applicability of the model, allowing for broader use in routine monitoring, telerehabilitation, or large-scale studies involving individuals with spinal cord injuries.

## 5. Conclusions

The marker-based motion analysis model developed in this study demonstrated initial stability and can be used to further investigate UE kinematics during wheelchair use in individuals with SCIs. The model exhibited good test–retest reliability; however, due to the small study population, further research is needed to validate these findings. The marker positions and joint calculations used to assess UE kinematics have potential clinical applications, including evaluating treatment effects and aiding in decision-making between different treatment modalities.

## Figures and Tables

**Table 1:** Demographics and clinical characteristics.

Characteristics	n(%)
**Gender**	
Male	9 (81)
Female	2 (19)
**Age** (yrs, Mean, SD)	52 (20)
**Injury type**	
Tetraplegia	1
Paraplegia	10
**AIS** *	
A	9 (81)
B	2 (19)

Data is presented as numbers and percentages unless otherwise noted. *: American Spinal Injury Association Impairment Scale.

**Table 2:** Marker Positions: BiL (bilateral)—two markers placed symmetrically on both sides of an anatomical landmark.

Segment	Number of Markers	Location
**Head**	4	Forehead, temple (BiL), rear head
**Shoulder**	3	Crest of acromion (BiL), crest of scapula (BiL)
**Trunk**	4	Jungular notch, xiphoid process, C7, T12
**Pelvis**	3	Backrest of chair, anterior superior iliac spine (BiL)
**Arm**	8	Medial humerus (BiL), epichondyle radii (BiL), epichondyle ulna (BiL), supra olecranon (BiL), radial styloid process (BiL), ulnar styloid process (BiL)
**Hand**	2	First metacarpophalangeal joint (BiL), fifth metacarpophalangeal joint (BiL)
**Wheelchair**	2	Drivewheel wheel hub (BiL), wheelhouse front wheel (BiL)

**Table 3:** Detailed statistical results presenting intraclass correlation coefficient (ICC) mean values for the test–retest assessment of the total range of motion across various parameters during wheelchair maneuvering at a self-selected speed. Statistically significant values are indicated by (*).

Total Range of Motion	ICC	*p*-Value	95% Confidence Interval of the ICC Agreement	
			Lower	Upper
Left Shoulder Sagittal	0.691	0.044 *	−0.214	0.918
Left Shoulder Frontal	0.781	0.011 *	0.212	0.941
Left Shoulder Rotation	0.782	0.012 *	0.194	0.941
Left Elbow Sagittal	0.849	0.004 *	0.418	0.960
Left Supination/Pronation	0.677	0.042 *	−0.181	0.913
Right Shoulder Sagittal	0.727	0.024 *	0.008	0.926
Right Shoulder Frontal	0.744	0.027 *	−0.022	0.932
Right Shoulder Rotation	0.596	0.090	−0.576	0.893
Right Elbow Sagittal	0.937	<0.001 *	0.775	0.983
Right Wrist Sagittal	0.952	0.000 *	0.819	0.987
Right Wrist Frontal	0.818	0.005 *	0.360	0.950
Right Supination/Pronation	0.897	0.001 *	0.628	0.972
Thorax to Wheelchair Sagittal	0.933	<0.001 *	0.704	0.983
Left Wrist Sagittal	0.421	0.213	−1.439	0.849
Left Wrist Frontal	0.589	0.102	−0.710	0.893

**Table 4:** Test–retest reliability of minimum range of motion during wheelchair maneuvering: Intraclass correlation coefficient (ICC) mean values across parameters at self-selected speeds (statistically significant values indicated by (*)).

Minimum Range of Motion	ICC	*p*-Value	95% Confidence Interval of the ICC Agreement	
			Lower	Higher
Left Shoulder Sagittal	0.965	0.000 *	0.870	0.990
Left Shoulder Frontal	0.914	0.001 *	0.643	0.977
Left Shoulder Rotation	0.800	0.012 *	0.215	0.947
Left Elbow Sagittal	0.748	0.015 *	0.130	0.931
Left Wrist Frontal	0.876	0.001 *	0.564	0.966
Left Supination/Pronation	0.898	0.001 *	0.627	0.972
Right Shoulder Sagittal	0.730	0.028 *	−0.041	0.928
Right Shoulder Frontal	0.869	0.001 *	0.537	0.964
Right Elbow Sagittal	0.719	0.029 *	−0.051	0.925
Right Wrist Sagittal	0.681	0.033 *	−0.084	0.912
Right Supination/Pronation	0.785	0.008 *	0.260	0.941
Thorax to Wheelchair Sagittal	0.907	0.000 *	0.673	0.975
Right Wrist Frontal	0.606	0.080	−0.488	0.894
Right Shoulder Rotation	0.399	0.234	−1.666	0.845
Left Wrist Sagittal	0.520	0.142	−0.956	0.874

**Table 5:** Intraclass correlation coefficients (ICCs) for test–retest reliability of maximum range of motion during wheelchair maneuvering at self-selected speeds (statistically significant values indicated by (*)).

Maximum Range of Motion	ICC	*p*-Value	95% Confidence Interval of the ICC Agreement	
			Lower	Upper
Left Shoulder Sagittal	0.726	0.019 *	0.076	0.924
Left Shoulder Rotation	0.968	<0.001 *	0.882	0.991
Left Elbow Sagittal	0.921	0.000 *	0.712	0.979
Left Wrist Sagittal	0.707	0.033 *	−0.098	0.922
Left Wrist Frontal	0.894	0.001 *	0.614	0.971
Left Supination/Pronation	0.948	0.000 *	0.810	0.986
Right Shoulder Sagittal	0.813	0.005 *	0.337	0.949
Right Shoulder Rotation	0.961	0.000 *	0.857	0.990
Right Elbow Sagittal	0.904	0.001 *	0.604	0.975
Right Wrist Sagittal	0.939	<0.1 *	0.770	0.984
Right Wrist Frontal	0.769	0.013 *	0.171	0.937
Right Supination/Pronation	0.754	0.013 *	0.162	0.932
Thorax to Wheelchair Sagittal	0.879	0.001 *	0.574	0.967
Right Shoulder Frontal	0.176	0.392	−2.941	0.791
Left Shoulder Frontal	0.417	0.220	−1.556	0.849

## Data Availability

The datasets used and/or analyzed during the current study are available from the corresponding author upon reasonable request.

## Abbreviations

The following abbreviations are used in this manuscript:

AIM	Automatic marker identification model
AIS	American Spinal Cord Injury Classification Scale: A scale for classifying if a spinal cord injury is complete or incomplete
3DOF	Three degrees of freedom: The assumption that a model has three degrees of freedom where motion is possible, around three axes, excluding translational motion
Hz	Hertz, the measurement for frequency
ICC	Intraclass correlation coefficient: A statistical measure of how well different measurements align with each other
IR	Infrared light
SCI	Spinal cord injury
